# Salutogenesis in Mexico and Latin America: Protocol For Scoping Review

**DOI:** 10.2196/83495

**Published:** 2026-04-16

**Authors:** Igor Martin Ramos Herrera, Rigoberto Antonio Cisneros Garcia

**Affiliations:** 1 Department of Public Health, University of Guadalajara, Regional Institute for Public Health Research, Sierra Mojada 950, Colonia Independencia, Guadalajara, 44340, Mexico, 52 (33) 1058 5200 ext 33902

**Keywords:** salutogenesis, Mexico, Latin America, public health, scoping review

## Abstract

**Background:**

The salutogenic model, introduced by Aaron Antonovsky, represents a fundamental paradigm shift from the traditional pathogenic orientation—which focuses on the etiology of disease—to a focus on the origins of health (salutogenesis). The core of this model is the Sense of Coherence, a global orientation that allows individuals to mobilize Generalized Resistance Resources to cope with the stressors of daily life. Although salutogenesis has been extensively researched in Europe and North America, its systematic application within diverse and multicultural contexts, marked by the inequity present in Mexico and Latin America, remains fragmented and insufficiently documented. There is a lack of clarity regarding how the model adapts to local realities, specifically in these countries.

**Objective:**

The main objective of this study is to conduct a scoping review to systematically map the extent, range, and nature of research activity on salutogenesis in Mexico and Latin America published between 2010 and 2026. Secondary objectives include identifying the theoretical frameworks employed, describing the psychometric properties of the instruments used to study the Sense of Coherence model in Spanish, English, and Portuguese, as well as determining research gaps to identify future guidelines for health promotion.

**Methods:**

This review will adhere to the methodological framework for scoping reviews developed by Arksey and O’Malley, refined by Levac et al, and the guidelines of the Joanna Briggs Institute. The protocol adheres to the PRISMA criteria for Scoping Reviews (PRISMA-ScR). A trilingual search (ie, English, Spanish, Portuguese) will be conducted in MEDLINE (PubMed), Scopus, Web of Science, PsycINFO, CINAHL, SciELO, LILACS, and Redalyc. Two independent reviewers will screen titles or abstracts and full-text articles, evaluating inter-rater reliability using Cohen Kappa coefficient. Data will be extracted using a piloted form and synthesized through descriptive numerical analysis and qualitative thematic analysis.

**Results:**

This protocol did not involve funding. The search strategy was finalized and preliminary searches were conducted in August 2025. Starting in February 2026, the study will begin the data integration phase. The final analysis is expected to be completed by August 2026, with submission for publication planned for December 2026.

**Conclusions:**

This scoping review will be the first to provide a holistic view of salutogenic research in Latin America. By synthesizing the evidence on how the model is operationalized in this region, the study will provide critical insights for the transition of public health systems from a curative model to one that fosters health assets, supporting the design of culturally congruent interventions.

## Introduction

### The Hegemony of the Pathogenic Paradigm

For centuries, biomedical sciences have been dominated by the pathogenic paradigm. This perspective asks, “Why do people get sick?” and focuses its resources on identifying pathogens, risk factors, and treating specific diseases [1]. While this approach has been responsible for monumental achievements in human survival—such as the eradication of smallpox and the management of infectious diseases—it inherently views health as a dichotomous variable (ie, the presence or absence of disease). Under this model, the goal of public health is to return the individual to a state of “normality” or homeostasis, largely defined by the absence of pathology [2]. However, this “deficit model” fails to explain why despite given the omnipresence of viruses, bacteria, stressors, and socioeconomic challenges, a significant portion of the population remains healthy and thrives.

### The Salutogenic Turn

#### Origins of Health

In the late 1970s, medical sociologist Aaron Antonovsky challenged this orthodoxy by introducing the concept of Salutogenesis (from the Latin *salus*=health, and the Greek *genesis*=origin). Antonovsky posited that chaos and stress are inherent to the human condition, not deviations from the norm. He proposed the “Health Ease/Dis-ease Continuum,” suggesting that individuals are not simply “healthy” or “sick,” but constantly moving along a spectrum between total health and breakdown [3]. The relevant question then shifts from “how do we treat disease?” to “how can we help a person move toward the health pole on the continuum, regardless of their current physiological state?”

#### Theoretical Core

The cornerstone of the salutogenic model is the Sense of Coherence (SOC). Antonovsky defined SOC as: “a global orientation that expresses the extent to which one has a pervasive, enduring though dynamic feeling of confidence.” This construct consists of three interrelated components:

1. Comprehensibility (Cognitive): The belief that stimuli deriving from internal and external environments are structured, predictable, and explicable.
2. Manageability (Instrumental): The perception that resources are available to meet the demands posed by these stimuli.
3. Meaningfulness (Motivational): The sense that these demands are challenges worthy of emotional investment and engagement.

Research suggests that people with a strong SOC are better able to identify and mobilize Generalized Resistance Resources (GRRs)—such as money, ego strength, social support, and cultural stability—to cope with stressors effectively, thereby maintaining or improving their health.

### The Latin American Context

#### A Unique Epidemiological Landscape

While the salutogenic model has been widely adopted in the Global North, specifically in Scandinavia, the United States, and Israel, its application in Latin America warrants specific investigation. For its part, the Latin American region is characterized by deep structural inequalities, rapid urbanization, and a polarized epidemiological profile where chronic non-communicable diseases (diabetes, hypertension, etc) coexist with infectious diseases and violence [4].

Furthermore, Latin America has a rich history of “Social Medicine” and “Collective Health,” which share philosophical roots with salutogenesis but have developed independently. Concepts such as *Buen Vivir* (Sumak Kawsay) in the Andean region emphasize harmony with the community and nature as a prerequisite for health, coinciding with the holistic nature of salutogenesis [5]. However, it is unclear to what extent Europe-derived salutogenic instruments (such as the SOC-29 or SOC-13 questionnaires) are culturally valid in these contexts, or whether they capture the nuances of Latin American resilience.

#### Rationale for a Scoping Review

Given the potential fragmentation of the literature in this region—scattered across psychology, public health, nursing, and sociology journals, and often published in Spanish or Portuguese—a classic systematic review focusing on intervention effectiveness would be premature. A scoping review is the most appropriate methodology to: (a) map the volume of research activity in the region, (b) clarify concepts and definitions used in the local literature, (c) identify research gaps (eg, lack of qualitative studies, absence of intervention studies), and (d) synthesize the evidence to inform the feasibility of a future full systematic review [6].

To the best of our knowledge, no comprehensive scoping review has specifically addressed the production of salutogenic knowledge in Mexico and Latin America. This protocol outlines the rigorous steps we will take to address this gap.

## Methods

### Study Design and Registration

This scoping review will be conducted according to the methodological framework originally proposed by Arksey and O’Malley [7] and later enhanced by Levac, Colquhoun, and O’Brien [8]. Additionally, we will follow the guidance provided by the Joanna Briggs Institute (JBI) Manual for Evidence Synthesis [9].

The methodology consists of five key stages: (1) identifying the research question, (2) identifying relevant studies, (3) study selection, (4) data charting, and (5) collating, summarizing, and reporting the results.

#### Stage 1: Identifying the Research Questions

We used the PCC (Population, Concept and Context) mnemonic recommended by the JBI to construct the research questions:

- Population human populations with no restriction on age, gender, or health status (eg, patients, students, health professionals) [10]
- Concept: Salutogenesis, SOC, Generalized Resistance Resources (GRRs)
- Context: Research conducted in Mexico, Central America, South America, and the Spanish/Portuguese-speaking Caribbean

#### Primary Research Question

What is the current state of scientific knowledge regarding salutogenesis and the SOC in Mexico and Latin America from 2010 to 2026?

#### Secondary Questions

What theoretical frameworks and definitions of salutogenesis are employed in Latin American research? Which versions of the SOC scale (SOC-13, SOC-29, or adapted versions) are most frequently used and have been validated for specific local populations? What are the main health outcomes (mental health, chronic disease management, health behaviors) associated with salutogenesis in the region? In what settings (clinical, educational, community, occupational) [11]. Is the model being applied?

### Stage 2: Identifying Relevant Studies

A comprehensive search strategy will be implemented to locate both published and gray literature. The search will be limited to documents published between January 1, 2010, and December 31, 2025, to capture the most recent developments and the evolution of the field over the last 15 years.

#### Information Sources

We will search the following electronic databases through access licenses provided by the University of Guadalajara, Mexico:

#### International Databases

MEDLINE (via PubMed), Scopus (Elsevier), Web of Science (Core Collection), PsycINFO (APA), and CINAHL (EBSCOhost).

#### Regional Databases

- SciELO (Scientific Electronic Library Online): covers a vast network of Latin American journals
- LILACS (Latin American and Caribbean Health Sciences Literature): The most comprehensive index of scientific and technical literature in the region
- Redalyc (Network of Scientific Journals of Latin America and the Caribbean, Spain and Portugal): A major open-access database of peer-reviewed journals from Latin America, Spain, and Portugal

#### Search Strategy

The search strategy will use a combination of controlled vocabulary (MeSH terms for PubMed, DeCS terms for LILACS/SciELO) and free-text keywords. The search will be conducted in English, Spanish, and Portuguese.

- **Keywords (English):** “Salutogenesis,” “Sense of Coherence,”“Sense of Coherence,” “Salutogenic,” “Generalized Resistance Resources,”“Generalized Resistance Resources,” “Latin America,” “Mexico,” “Brazil,” “Argentina,” “Colombia,” “Chile,” “Peru,” “Central America,” “South America.”
- **Keywords (Spanish):** “Salutogénesis,” “Sentido de Coherencia,” “Modelo Salutogénico,” “Recursos Generales de Resistencia,” “América Latina,” “México.”
- **Keywords (Portuguese):** “Salutogênese,” “Senso de Coerência,” “Recursos Gerais de Resistência,” “América Latina.”

A draft of the search strategy for PubMed is provided in Multimedia Appendix 1.

#### Gray Literature Strategy

To minimize publication bias, we will search for gray literature in:

**Google Scholar (reviewing the first 200 results):** A broad multidisciplinary search engine that retrieves scholarly literature across formats, useful for capturing a wide range of academic sources

**Institutional repositories of major Latin American universities (e.g., UNAM, University of São Paulo, University of Guadalajara):** University-based repositories that provide access to theses, dissertations, articles, and other academic outputs produced by institutional researchers and students

**ProQuest Dissertations and Theses:** A major database of graduate theses and dissertations, offering access to extensive scholarly work from universities worldwide

### Stage 3: Study Selection

This process will be managed using Covidence (Veritas Health Innovation) or Rayyan software to ensure transparency and blinding.

#### Inclusion Criteria

- Study type: Primary empirical studies (quantitative, qualitative, mixed), systematic reviews, meta-analyses, and theoretical articles proposing specific adaptations for the region [12]
- Language: Spanish, English, or Portuguese
- Topic: The study must explicitly address salutogenesis or measure the SOC as a primary variable
- Geography: Conducted in Latin American countries

#### Exclusion Criteria

- Editorials, commentaries, and opinion pieces without original data or theoretical contribution
- Conference abstracts where the full text is not available
- Studies conducted on Latin American immigrants residing outside the region (eg, in the USA or Spain), as the environmental context differs from the scope of this review

### Screening Process

#### Calibration

Before starting the formal screening, the two reviewers (IMRH and RACG) will independently evaluate a random sample of 50 citations. We will calculate inter-rater reliability using Cohen’s Kappa coefficient. If Kappa<0.75, reviewers will discuss discrepancies and refine the inclusion criteria.

#### Level 1 Screening (Title and Abstract)

Two reviewers will independently examine all retrieved citations.

#### Level 2 Screening (Full Text):

The full text of potentially eligible articles will be retrieved and evaluated independently. Reasons for exclusion will be recorded for each article rejected at this stage. Discrepancy Resolution: Any disagreement will be resolved through discussion or by consulting a third senior reviewer if consensus cannot be reached.

### Stage 4: Data Charting

A data extraction form will be developed in Microsoft Excel. The form will be iteratively updated; the two reviewers will pilot test the form on a sample of 5‐10 studies to ensure it captures all necessary nuances.

The data items to be extracted include:

1. Bibliometric data: Author(s), year, title, journal, country of origin, publication language
2. Methodological data: Study design (eg, cross-sectional, cohort, ethnographic), sample size, sampling method, study duration
3. Population characteristics: Age group (children, adolescents, adults, elderly), gender distribution, socioeconomic status, health status (healthy vs clinical population)

#### Salutogenic Variables

The methodology framework begins with a clear definition of salutogenesis tailored to the study’s context. To quantify the SOC, researchers utilized established instruments such as the SOC-13, SOC-29, SOC-L, or the Family SOC scale. Furthermore, the study details the reliability coefficients (Cronbach α) specifically calculated for the local population to ensure psychometric integrity. Finally, the analysis incorporates additional salutogenic constructs, specifically Generalized Resistance Resources (GRRs).

#### Main Findings

The main findings included associations between SOC and health outcomes, as well as the main themes identified in qualitative studies. Conclusions included the authors’ recommendations for policy or practice.

### Stage 5: Collating, Summarizing, and Reporting Results

We will synthesize the data using a hybrid thematic framework synthesis. This approach allows for a systematic link between our research questions and the evidence mapping by combining deductive and inductive coding. The process will follow the framework analysis method proposed by Ritchie and Spencer [13] and the JBI methodological guidance for evidence synthesis [14].

#### Deductive Coding

An initial framework will be established using categories derived from the study objectives, such as “Theoretical Frameworks” and “SOC Scale Variations.” These represent the “expected themes” identified in the pilot search.

#### Inductive Refinement

Following the reflexive thematic analysis phases of Braun and Clarke [15], reviewers will read the data to identify emerging codes that capture regional nuances, such as the transition from individual to collective resilience or the integration of *Buen Vivir* [16].

#### Mapping and Synthesis

The final narrative report will integrate numerical descriptions with these themes to provide a holistic view of the salutogenic paradigm in Mexico and Latin America.

## Results

### Project timeline

The project’s timeline began with the conceptualization of the study in January 2025. This was followed by a preparatory phase from August to December 2025, which focused on protocol development and team training. The operational stages for 2026 commenced with database searches and duplicate removal in February and March, leading into the title and abstract screening in April. Looking ahead, the full-text review is projected for May and June, followed by data extraction and synthesis in July. The project is expected to conclude with the reporting phase, involving manuscript writing and submission, scheduled between August and December 2026.”

### Preliminary Search Results

**Figure 1.**
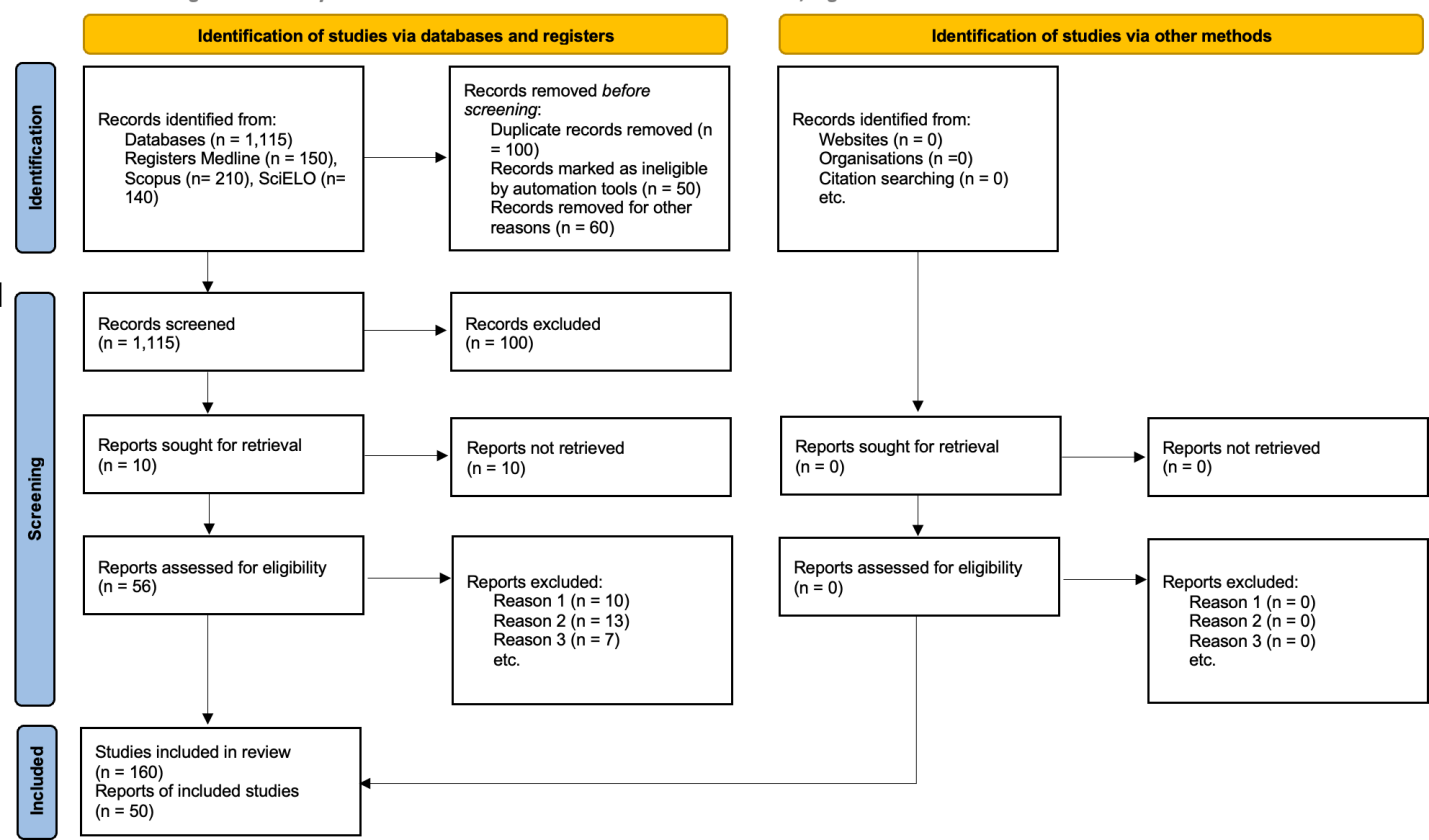
PRISMA diagram (PRISMA Extension for Scoping Reviews) [18].

An initial pilot search in PubMed using the term “Salutogenesis AND Mexico” yielded 109 results, while “Salutogenesis AND Latin America” yielded 1150 results. This confirms the feasibility of the review but highlights the need for a trilingual search strategy to capture the full breadth of the literature. The protocol has been prepared following the PRISMA Extension for Scoping Reviews (PRISMA-ScR) checklist [17] and the preliminary selection process is shown in Figure 1.

## Discussion

### Maturation of the Paradigm and the Global-Local Gap

This protocol represents a critical step in the maturation of health promotion research in Latin America [19]. While salutogenesis has been extensively reviewed globally—with a strong predominance of studies in Global North contexts—the translation of this paradigm to Latin American socioeconomic and epidemiological realities remains fragmented. Foundational systematic reviews have mapped the impact of the SOC on specific health outcomes [1]; however, there remains a persistent need for a comprehensive synthesis addressing how the model is operationalized in contexts of high structural inequality. By consolidating literature from regional databases such as LILACS and Redalyc, this study aims to bridge a critical gap, retrieving evidence that is often rendered invisible in Anglophone-centric reviews.

### Theoretical Implications: The Tension Between Individualism and Collectivism

We hypothesize that this review will reveal a fundamental epistemological tension between the individualistic genesis of the SOC construct and the inherently collectivistic nature of Latin American cultures. The protocol seeks to clarify whether the use of standard scales (SOC-29, SOC-13) has been uncritical or if there is a transition toward culturally adapted instruments that capture family and community resilience. This analysis is essential for theoretical development, as it allows for testing the universality of Antonovsky’s constructs under regional worldviews such as *Buen Vivir* (Sumak Kawsay) [20,21], suggesting that health in the Global South is not merely an individual psychological trait but a socio-ecological process of collective management.

### Implications for Practice and Public Policy

From a practical standpoint, the protocol is oriented toward transforming the traditional deficit-based model into an asset-based one. If the review confirms that a robust SOC is associated with better management of chronic conditions (such as diabetes in resource-limited settings), it will strengthen the argument for integrating salutogenic interventions into primary care [22]. Specifically, the findings could support the implementation of “social prescribing,” connecting patients with community assets and support networks that bolster their comprehensibility, manageability, and meaningfulness. At a policy level, this study seeks to align with the PAHO Strategy and Plan of Action on Health Promotion 2019‐2030, providing evidence to shift from a “war on disease” toward governance that generates health through intersectoral interventions [23,24].

### Design Strengths and Limitations

The main strength of this protocol lies in its trilingual search strategy and methodological rigor based on JBI and PRISMA-ScR guidelines. By including regional databases, representativeness is ensured, challenging global publication biases. However, it is acknowledged as a limitation that, as a scoping review, a formal assessment of the risk of bias of the included studies will not be conducted; instead, the priority will be an exhaustive mapping of the state of the art in the region.

The results will be disseminated through publication in a high-impact, peer-reviewed indexed journal. Additionally, we plan to create a brief for policymakers and present the findings at the Mexican Public Health Congress to ensure the translation of knowledge into local practice.

## Conclusion

The transition toward a health system that truly promotes well-being requires solid theoretical foundations [25]. This protocol establishes the roadmap to assess whether salutogenesis can be that foundation in Latin America [26]. By mapping the existing evidence, it not only identifies academic gaps but also takes a step toward the decolonization of health knowledge, validating how our populations build health amidst adversity [27].

## Supplementary material

10.2196/83495 Multimedia Appendix 1 Search Strategy.

10.2196/83495 Checklist 1 PRISMA-ScR checklist.

## Abbreviations

GRR: Generalized Resistance Resources
PRISMA-ScR: PRISMA Extension for Scoping Reviews
SOC: Sense of Coherence
