# Supplementary material for: Salutogenesis in Mexico and Latin America: Protocol For Scoping Review
Source: JMIR Res Protoc. 2026 Apr 16;15:e83495. doi: 10.2196/83495 (PMC13086258; doi:10.2196/83495)
Supplement: Multimedia Appendix 1 [file resprot-v15-e83495-s001.docx]

Search Strategy

| Search Step | Consultation | Results (Approx.) |
| --- | --- | --- |
| **#1** | ("Salutogenesis"[MeSH Terms] OR "Salutogenesis"[Title/Abstract] OR "Sense of Coherence"[Title/Abstract] OR "Generalized Resistance Resources"[Title/Abstract] OR "SOC-13"[Title/Abstract] OR "SOC-29"[Title/Abstract]) | ~4,500 |
| **#2** | ("Latin America"[MeSH Terms] OR "Mexico"[MeSH Terms] OR "Brazil"[MeSH Terms] OR "South America"[MeSH Terms] OR "Central America"[MeSH Terms] OR "Caribbean Region"[MeSH Terms] OR "Colombia"[Title/Abstract] OR "Argentina"[Title/Abstract] OR "Chile"[Title/Abstract] OR "Peru"[Title/Abstract] OR "Venezuela"[Title/Abstract] OR "Ecuador"[Title/Abstract] OR "Guatemala"[Title/Abstract] OR "Cuba"[Title/Abstract] OR "Bolivia"[Title/Abstract] OR "Dominican Republic"[Title/Abstract] OR "Honduras"[Title/Abstract] OR "Paraguay"[Title/Abstract] OR "El Salvador"[Title/Abstract] OR "Nicaragua"[Title/Abstract] OR "Costa Rica"[Title/Abstract] OR "Panama"[Title/Abstract] OR "Uruguay"[Title/Abstract]) | ~1,500,000 |
| **#3** | #1 AND #2 | ~150 |
| **#4** | Filtrar #3 by date: 2010/01/01 – 2026/03/31 | ~120 |
| **#5** | Filtrar #4 by language: English, Spanish, Portuguese | ~115 |
